# Dietary Zinc Intake and Its Association with Metabolic Syndrome Indicators among Chinese Adults: An Analysis of the China Nutritional Transition Cohort Survey 2015

**DOI:** 10.3390/nu10050572

**Published:** 2018-05-08

**Authors:** Yun Wang, Xiao-Fang Jia, Bing Zhang, Zhi-Hong Wang, Ji-Guo Zhang, Fei-Fei Huang, Chang Su, Yi-Fei Ouyang, Jian Zhao, Wen-Wen Du, Li Li, Hong-Ru Jiang, Ji Zhang, Hui-Jun Wang

**Affiliations:** National Institute for Nutrition and Health, Chinese Center for Disease Control and Prevention, Beijing 100050, China; wangyunn@aliyun.com (Y.W.); jiaxf@ninh.chinacdc.cn (X.-F.J.); zzhangb327@aliyun.com (B.Z.); wangzh@ninh.chinacdc.cn (Z.-H.W.); zhangjg@ninh.chinacdc.cn (J.-G.Z.); huangff@ninh.chinacdc.cn (F.-F.H.); suchang@ninh.chinacdc.cn (C.S.); ouyyf@ninh.chinacdc.cn (Y.-F.O.); zhaojian131023@163.com (J.Z.); duww@ninh.chinacdc.cn (W.-W.D.); lili@ninh.chinacdc.cn (L.L.); jianghr@ninh.chinacdc.cn (H.-R.J.); zhangji@ninh.chinacdc.cn (J.Z.)

**Keywords:** dietary zinc, Chinese adults, food source, metabolic syndrome

## Abstract

The dietary zinc consumed in Chinese households has decreased over the past decade. However, the national dietary zinc intake in the last five years has seldom been investigated. Using data from 12,028 participants 18 to 64 years old (52.9% male) in the China Nutritional Transition Cohort Survey (CNTCS) 2015, we describe the intake of dietary zinc and the contributions of major foods and we examine the relationship between the level of dietary zinc intake and metabolic syndrome indicators, including blood pressure, fasting glucose, and triglycerides (TG), in Chinese adults. We assessed dietary zinc intake using 24 h recalls on three consecutive days. The mean daily dietary zinc intake for all participants was 10.2 milligrams per day (males 11.2 mg/day, females 9.4 mg/day, *p* < 0.001). The mean daily dietary zinc density for all participants was 5.2 mg/day per 1000 kilocalories. Among all participants, 31.0% were at risk of zinc deficiency, with dietary zinc intakes of less than the Estimated Average Requirement (EAR) (males 49.2%, females 14.8%, *p* < 0.050), and 49.9% had adequate dietary zinc intakes, equal to or greater than the recommended nutrient intake (RNI) (males 30.7%, females 67.0%, *p* < 0.050). We found substantial gender differences in dietary zinc intake and zinc deficiency, with nearly half of the men at risk of zinc deficiency. Males of younger age, with higher education and incomes, and who consumed higher levels of meat, had higher zinc intakes, higher zinc intake densities, and higher rates of meeting the EAR. Among all participants, grains, livestock meat, fresh vegetables, legumes, and seafood were the top five food sources of zinc, and their contributions to total dietary zinc intake were 39.5%, 17.3%, 8.9%, 6.4%, and 4.8%, respectively. The groups with relatively better dietary zinc intakes consumed lower proportions of grains and higher proportions of livestock meat. For males with adequate dietary zinc intake (≥RNI), TG levels increased by 0.219 millimoles per liter (mmol/L) compared with males with deficient dietary zinc intake (<EAR). For females in the ≥RNI group, diastolic blood pressure decreased by 0.963 millimeters of mercury (mmHg) and fasting glucose decreased by 0.187 mmol/L compared with females in the <EAR group; in addition, TG increased by 0.097 mmol/L in females in the ≥RNI group and by 0.120 mmol/L in females in the equal to or greater than the EAR and less than the RNI (EAR-RNI) group compared with females in the <EAR group. Adequate dietary zinc was associated with reduced diastolic blood pressure and fasting glucose levels in female Chinese adults, but with raised TG levels in all Chinese adults. We recommend strengthened nutrition interventions for Chinese males and lower socioeconomic subgroups.

## 1. Introduction

Inadequate dietary zinc intake is widespread in the world’s population, especially in developing countries, where staple diets are predominantly plant-based, and consumption of animal source foods, such as red meat, poultry, and fish, is often modest because of economic, cultural, and religious constraints [1]. At present, China, as the largest developing country, is experiencing critical changes in its nutrition and health along with its rapid economic development. The dietary structure and the population’s consumption of food, energy, and nutrients are changing significantly [2]. Studies have showed that the intake of dietary zinc in Chinese households has decreased over the past decade [3,4,5,6,7,8], but few studies have examined Chinese dietary zinc intake in the last five years. Using the most recent data from 2015, we analyzed the dietary zinc intake of Chinese adults, assessed the prevalence of zinc deficiency, determined the main food sources of zinc, and examined the impact of the socioeconomic status on dietary zinc intake. 

Zinc is a trace element essential to many metabolic processes and is important for growth and development, immunity, neurological functions, and reproduction [9,10,11,12]. It is present in many enzymes, such as alkaline phosphatase, copper/zinc superoxide dismutase, nitric oxide synthase, neutral endopeptidase, and angiotensin-I-converting enzyme [13,14]. In addition, studies have documented that zinc acts as an antioxidant, has membrane-stabilizing properties, blocks apoptotic cell death, and is essential for endothelial integrity [15,16]. Therefore, zinc plays a substantial role in the prevention of metabolic syndrome [17], including atherogenic dyslipidemia, hyperglycemia, insulinemia, and elevated blood pressure, through the inhibition of proinflammatory cytokine expression, which suppresses reactive oxygen species (ROS) production, protecting against oxidative stress damage. In addition to ROS neutralization, zinc participates in glucose and lipid metabolism. Numerous studies have showed that zinc supplements improve blood pressure, glucose, and lipid levels. However, the results of previous studies on the association between zinc status and metabolic indicators are controversial [18], as they used serum zinc concentrations derived from patient populations and experiments. Despite the clinical significance of zinc deficiency, there is no established method or biomarker to reliably evaluate the zinc status [19], although serum zinc is widely used in most studies. To the best of our knowledge, few studies have addressed the relationship between dietary zinc intake and metabolic syndrome indicators in epidemiological studies. Accordingly, in addition to describing dietary zinc intake levels in Chinese adults, we examined the relationship between dietary zinc intake and metabolic indicators, including blood pressure, fasting glucose, and triglycerides (TG) in that population.

## 2. Methods

### 2.1. Study Population

Our study used data from the China Nutritional Transition Cohort Survey (CNTCS), a nationally financed project of the National Institute for Nutrition and Health, Chinese Center for Disease Control. The CNTCS is a longitudinal tracking survey based on the ongoing China Health and Nutrition Survey (CHNS), a collaboration between the National Institute for Nutrition and Health, Chinese Center for Disease Control, and the University of North Carolina at Chapel Hill [20]. In 2015, the CHNS added Zhejiang, Yunnan, and Shaanxi to the original 12 provinces (districts/cities) (Beijing, Liaoning, Shanghai, Jiangsu, Shandong, Heilongjiang, Henan, Hunan, Hubei, Chongqing, Guizhou, Guangxi).

In our study, we used a stratified multistage cluster random sampling method. We included CNTCS participants 18 to 64 years old who had complete demographic and dietary intake data. We excluded those with implausible energy intakes (<500 kilocalories per day (kcal/day) or >5000 kcal/day), pregnant women, and nursing mothers. We excluded patients with disease history of hypertension, diabetes, and cardiovascular diseases from the data collected by questionnaires. Our study population totaled 12,028 participants.

The Ethics Committee of the National Institute for Nutrition and Health, Chinese Center for Disease Control, approved all procedures involving human subjects. All participants gave their written informed consent.

### 2.2. Data Collection

Trained interviewers collected dietary data with three-day, 24-h diet retrospective surveys. The interviewers used standard forms for the dietary recalls with food models and picture aids in household interviews. For individual dietary intake, each household member reported all foods consumed (meals and snacks) at home and away from home in the previous 24 h for three consecutive days. We used the Chinese Food Composition Table [21] to calculate individual daily intake of nutrients and daily energy intake (kcal) for the foods collected in the dietary data. The survey also collected individual lifestyle information, including smoking and drinking status, in a questionnaire during the household interview. 

The interviewers measured weight without shoes and in light clothing to the nearest 0.1 kilogram (kg) and height to the nearest 0.1 cm without shoes. We calculated the body mass index (BMI) as weight (kg) divided by height (meters) squared. The interviewers collected blood samples from all participants after an overnight fast of at least 10 h and measured fasting glucose and TG. We measured blood pressure three times and used the average.

### 2.3. Assessment of Dietary Zinc Intake

We used the Estimated Average Requirement (EAR) and the recommended nutrient intake (RNI) in the Dietary Reference Intake guide for zinc in Chinese adults [22] to divide dietary zinc intake into three levels: less than the EAR (<EAR), equal to or greater than the EAR and less than the RNI (EAR-RNI), and equal to or greater than the RNI (≥RNI). The male EAR is 10.4 milligrams per day (mg/day), the male RNI is 12.5 mg/day, the female EAR is 6.1 mg/day, and the female RNI is 7.5 mg/day. We defined dietary zinc intakes that were <EAR as deficient and dietary zinc intakes that were ≥RNI as adequate zinc intakes.

### 2.4. Grouping Standards

We divided participants into two age groups, i.e., 18–49 and 50–64 years, and four education level groups, i.e., primary/below, middle school, high school, university/above. We categorized incomes into tertiles, i.e., low, medium, and high. We divided the regions into city, suburb, county, and rural according to China’s administrative classifications. We divided the eastern (Beijing, Liaoning, Shanghai, Jiangsu, Zhejiang, Shandong), central (Heilongjiang, Henan, Hubei, Hunan), and western (Chongqing, Guizhou, Yunnan, Shaanxi, Guangxi) areas according to China’s economic zones [23] into developed, medium-developed, and underdeveloped areas, respectively. 

### 2.5. Statistical Analysis

We used SAS version 9.2 (SAS Institute, Inc., Cary, NC, USA) for data cleaning and analysis. We employed analysis of variance (ANOVA) to test the differences in dietary zinc intake and dietary zinc density between genders and ages. With linear trend tests, we tested the trends in dietary zinc intake and dietary zinc density between education levels, income levels, regions, and areas. We used a chi-square test for the differences in the distribution of dietary zinc intake levels and the proportions of dietary zinc food sources between genders and ages. We conducted the Cochran–Armitage trend test to test the trends of dietary zinc intake levels and the proportions of dietary zinc food sources between education levels, income levels, regions, and areas. We used linear regression models to study the effects of dietary zinc intake levels on metabolic syndrome indicators.

## 3. Results

### 3.1. Characteristics of the Study Population

Of our 12,028 subjects, 47.1% (*n* = 5665) were male. The males had higher education and income levels than the females. Intakes of energy, protein, carbohydrate, fat, and sodium were significantly higher in males than in females, as were smoking and drinking rates, BMI, systolic blood pressure, diastolic blood pressure, fasting glucose, and TG levels (*p* < 0.001) (Table 1).

### 3.2. Dietary Zinc Intake

The average dietary zinc intake for all participants was 10.2 mg/day. The dietary zinc intake differences between gender, age, region, education level, income level, and area were statistically significant. The dietary zinc intake was higher in males than in females and also higher in the 18–49 age group than in the 50–64 age group. The intake increased gradually with regional development and with higher education. The high-income group and the western area group consumed the highest levels of zinc. The same age, region, education level, and income level differences were apparent in males and females but not the area difference. 

The average dietary zinc density for all participants was 5.2 mg/day per 1000 kcal. The results showed no differences in dietary zinc density between the genders, while differences in age, region, education level, income level, and area were statistically significant. The dietary zinc density was higher in the 18–49 age group than in the 50–64 age group. The dietary zinc density increased gradually with regional development, higher education, and higher income. Those in the eastern area showed the highest dietary zinc density. Males and females showed the same age, region, education, income, and area differences (Table 2). 

### 3.3. Dietary Zinc Intake Level

We further investigated the distribution of dietary zinc intake levels and found that 31.0% of all participants consumed a level <EAR and 49.9% consumed a level ≥RNI. The proportion of participants with dietary zinc intake levels ≥RNI was significantly higher in females than in males, while the proportion with zinc intake levels <EAR was higher in males. 

For males and females, the proportions of participants with zinc intakes level ≥RNI were higher among those 18–49 years old, those in the most developed cities, those with highest education levels (university and above), those with the highest income levels, and those in the western area. On the contrary, the proportions of participants with zinc intake levels <EAR were lower in all of those groups (Table 3).

### 3.4. Food Sources of Dietary Zink

According to the food classifications from the Chinese Food Composition Table [14], grains, livestock meat, fresh vegetables, legumes, and seafood were the top five food sources of zinc for all participants, and their contributions to total dietary zinc intake were 39.5%, 17.3%, 8.9%, 6.4%, and 4.8%, respectively. The five groups together accounted for 76.9% of the total dietary zinc intake, and grains and livestock meat accounted for 56.8% of the total. 

Zinc intakes from grains, livestock meat, and seafood were significantly lower in females than in males, while intakes from vegetables and legumes were higher in females than in males. Zinc intakes from grains, vegetables, and legumes were significantly lower in the 18–49 age group than in the 50–64 group, while the intake from livestock meat was higher in the 18–49 group than in the 50–64 group. The zinc contribution rates from grains, vegetables, and legumes decreased with more regional development, higher education, and higher income, while the rates from livestock meat and seafood increased. We found the largest differences in food sources of dietary zinc in grains and the second largest in livestock meat. The zinc contribution from grains was 13.0% higher in the rural group than in the city group, i.e., 44.2% versus 31.2%, respectively, and the contribution from livestock meat was 13.0% higher in the western area than in the central area, i.e., 30.4% versus 17.4%, respectively (Table 4).

**Figure 1:**
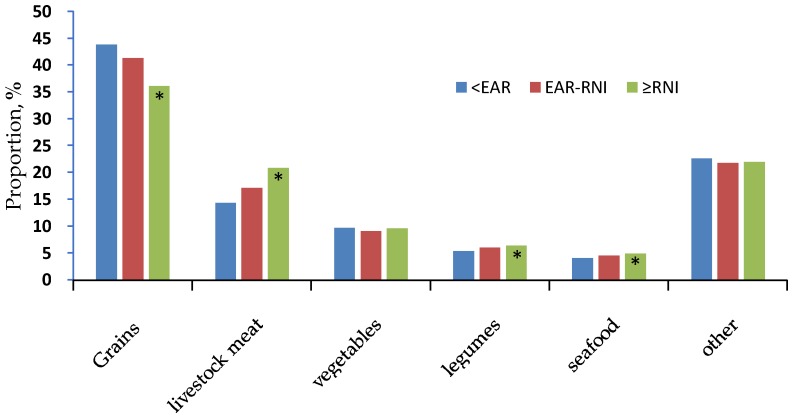
Food sources of dietary zinc by levels of zinc intake. The asterisk * indicates a significant difference from the Cochran–Armitage trend test, *p* < 0.001.

Comparing the dietary zinc intakes corresponding to <EAR, EAR-RNI, and ≥RNI, we found that dietary zinc from grains decreased, while those from livestock meat, legumes, and seafood increased from EAR to RNI. Zinc from grains decreased by 7.7% in the ≥ RNI level compared to the < EAR level, while zinc from livestock meat increased by 6.5% (Figure 1). 

### 3.5. Relationship between Dietary Zinc Intake and Metabolic Syndrome Indicators 

Using linear regression models, we studied the effect of dietary zinc intake on systolic blood pressure, diastolic blood pressure, fasting glucose, and TG in both genders after adjusting for age, region, education, income, area, intake of energy, protein, carbohydrate, fat, and sodium, alcohol consumption, and smoking. We considered the level <EAR as the reference. For males, TG increased by 0.219 mmol/L in the ≥RNI level (Table 5). For females, the effects of dietary zinc intake on diastolic blood pressure, fasting glucose, and TG were statistically significant. Diastolic blood pressure decreased by 0.963 mmHg in the ≥RNI level, fasting glucose decreased by 0.187 mmol/L in the ≥RNI level, and TG increased 0.097 mmol/L in the ≥RNI level and 0.120 mmol/L in the EAR-RNI level (Table 6).

## 4. Discussion

This study found that, in 2015, the average dietary zinc intake of Chinese adults in 15 provinces was 10.2 mg/day, which is lower than the results from the China National Nutrition and Health Survey that measured 11.3 mg/day in 2002 and 10.7 mg/day in 2010–2012 [24]. This study found that the average dietary zinc density was 5.2 mg/day per 1000 kcal, higher than the results from the China National Nutrition and Health Survey in 2002 (5.1–5.2 mg/day per 1000 kcal in urban residents and 4.7–4.8 mg/day per 1000 kcal in rural residents) [25]. The comparison of the above results suggests that, over time, up to 2015, Chinese dietary zinc intake was on a declining trend and Chinese dietary zinc density was on the rise. Our study found that 31.0% of Chinese adults consumed the dietary zinc at a level <EAR, which was slightly lower than the 35.6% from the China National Nutrition and Health Survey in 2010–2012 [8]. Our results indicate that the dietary zinc intake of nearly half of the male population was <EAR. In contrast, the dietary zinc intake of women was better, with 85.2% of women reaching the EAR, and 67.0% reaching the RNI.

In the past decade or so, China’s food structure has changed greatly. Consumption of grains, vegetables, and other plants has decreased, and consumption of animal source foods, such as livestock and poultry, has significantly increased [26]. In 2002, 51.9% of dietary zinc was from grains, and 13.5% was from meat [7]. By 2015, the consumption of grains had decreased by 13.1%, the consumption of meat had increased by 5.2%, and the average intake of dietary zinc had decreased. The reason may be related to the changes in food sources in the past 10 years. Meat consumption did not increase as much as grain consumption decreased, which might have improved dietary zinc density. However, the Chinese total food and energy intake was on the decline [8], so zinc intake showed a downward trend. 

Gender, age, education, income, region, and area differences may affect the dietary zinc intake. Men consumed more zinc than women. Since we found no difference in dietary zinc density between the genders, the dietary zinc intake differed from the total energy intake. The dietary zinc intake in the 50–64 age group was lower than in the 18–49 age group, possibly because of the lower dietary zinc density and total energy intake in the 50–64 group. Education, income, and regional development might have positive effects on dietary zinc intake. Higher levels of education, income, and regional development corresponded to higher dietary zinc intakes and higher zinc densities, but the study also found that the highest levels of zinc density were in the least developed western region. This result might be related to the higher meat consumption in that region, where 30.4% of dietary zinc intake was from livestock meat.

The zinc intake differences reflect the rates of dietary zinc deficiency. The higher the dietary zinc intake, the higher the rate of meeting the EAR and the RNI. The Chinese zinc evaluation standards for males and females are not the same, so although the mean value showed a difference of only 1.8 mg/day between the genders, we found a huge gap in the rates of dietary zinc deficiency. Only 14.8% of women did not reach the EAR, while nearly half of men did not reach it. In addition, 67.0% of women reached the RNI, while only 30.7% of men reached it, less than half the rate for women.

Food sources affected dietary zinc intake. As dietary zinc intake increased, the proportion of grain intake decreased, and the proportion of meat intake increased significantly. Therefore lower grain and higher livestock meat consumption characterizes a relatively good dietary zinc status for adults in China.

In comparison, in the United States, an economically developed country, the dietary zinc intakes for males and females over the age of 19 were 14.2 and 9.8 mg/day, respectively, in NHANES 2009–2010, and the rates of zinc deficiency were 11.0% and 17.0% for males and females, respectively, in HNANES 2001–2002 [27]. Chinese women’s zinc intakes and inadequacies in this study were comparable to those of American women, but Chinese men’s zinc intakes and inadequacies in this study were far from those of American men. The top three food sources of dietary zinc in the United States in HNANES 2003–2006 were meats, grains, and dairy products, accounting for 35.0%, 15.0%, and 13.0%, respectively [28,29,30]. The rough proportions of dietary zinc from grains and meats in China in this study were just the opposite, and dairy was a big difference. China’s dairy intake was low, so dairy products were not an important source of zinc. Although the average dietary zinc intake in China was lower than that in the United States, it was comparable to those in Canada [31] and New Zealand [32] and higher than those in the United Kingdom [33,34] and Chile [35]. However, the adequacy of zinc intake depends not only on the amount of zinc but also on its bioavailability. People consuming a diet that provides marginal zinc may not absorb an adequate amount of zinc if they also consume foods high in phytates. The average phytate intake for people in China (1186 mg/day) [22,36] was higher than that in western countries, possibly because of the higher consumption of grains and lower consumption of meats and dairy products in China. Although Chinese dietary zinc intake was comparable with that of some western countries, Chinese dietary zinc absorption was not necessarily the same. By the way, it seems that the proportion of foods high in phytate was more prevalent in rural regions, where the zinc intake was also lower. This would mean that bioavailability was particularly low in the geographic population with the lowest intake, which may aggravate the problem of zinc deficiency in these areas.

Zinc is highly significant in the pathogenesis of metabolic syndrome, which suggests that zinc supplementation would have a positive effect in reducing metabolic syndrome. However, the results of previous studies on the association between zinc and metabolic indicators are controversial. For example, it has been reported that zinc may participate in blood pressure regulation and in the pathogenesis of hypertension [37]. Inverse relationships between blood pressure and dietary zinc and serum zinc concentration have been documented in hypertensive subjects [38,39], but some studies have reported that zinc does not change the blood pressure in rats [40,41] or humans [42]. It also has been suggested that zinc deficiency may be an important risk factor for type 2 diabetes mellitus [43]. Several studies have observed a decreased concentration of zinc in diabetic patients compared to healthy people [44,45,46,47,48,49,50,51], and others have noticed a relation between inadequate zinc intake and raised insulin concentrations in blood in adolescents [52]. Some recent studies showed that zinc supplementation improved glucose metabolism and insulin sensitivity in diabetic patients [53,54,55,56,57]. However, other studies did not confirm the association between zinc and glucose metabolism and insulin resistance [58,59]. Numerous studies have indicated an association between serum zinc levels and lipid metabolism [60]. Clinical and experimental studies have reported that zinc supplementation decreased total cholesterol, low-density lipoprotein cholesterol, and TG and increased high-density lipoprotein cholesterol [53,61]. In other studies, zinc deficiency exacerbated hepatic lipid metabolism, while zinc supplementation increased hepatocyte activity and improved lipid metabolism in the liver [62,63]. By contrast, E. Weigand and J. Egenolf [64] have showed that moderate zinc deficiency did not alter lipid concentration and fatty acid composition in the livers of rats fed a high-fat diet. Moreover, short-term zinc supplementation in obese patients decreased weight and TG levels without significant changes in lipid and glucose profiles [58,65,66], and, elsewhere, serum zinc levels in men were positively associated with elevated TG [67].

In contrast with most of the above results, where the association of serum zinc status with metabolic syndrome were based on experimental or patient studies, our study emphasizes the effects of dietary zinc intake on metabolic syndrome indicators based on a health cohort. For females, the group with adequate dietary zinc intake (≥RNI) showed lower diastolic blood pressure and fasting glucose than the reference group (<EAR). Our systolic blood pressure difference was not statistically significant, although the results from a study showed that dietary zinc intake was inversely associated with systolic blood pressure in young obese Korean women [68]. For males and females, the dietary zinc intake was positively associated with TG. Our study demonstrates that adequate dietary zinc intake was associated with reduced diastolic blood pressure and fasting glucose levels. However, we saw a rise in TG in the groups with adequate dietary zinc intakes, which seemed inconsistent with the improvement in lipid metabolism. The relationship between dietary zinc intake and serum zinc concentration was inconsistent because of high heterogeneity [69], and the improvement in lipid metabolism was contradictory. Nevertheless, this study found that adequate dietary zinc was associated with reduced blood pressure and fasting glucose, as did previous studies of serum zinc levels. Further studies should investigate the role of serum zinc and urine zinc concentrations on metabolic syndrome, and further studies of gender-specific influences are needed to evaluate the effects of dietary zinc intake on metabolic syndrome.

This study has several limitations. The three-day, 24 h food intake recall may not reflect long-term dietary habits. Eating out is another source of potential measurement errors [70]. Another limitation is that the study did not take into account zinc from dietary supplements. As the use of dietary supplements is less widespread in China (10.3% in urban residents, 2.9% in rural residents) [71] than in the United States (39.5% overall) [72], we assumed that our underestimation of zinc intake was small. Although we excluded patients affected by hypertension, diabetes, and cardiovascular diseases from the data collected by questionnaires, the questionnaires could not rule out undetected participant with metabolic disease which might have caused minor errors.

## 5. Conclusions

In conclusion, we found a substantial gender difference in dietary zinc intake and zinc deficiency. Nearly half of the men were at risk of zinc deficiency, whereas very few women were at risk. Grains and livestock meat were the main food sources of dietary zinc. The local culture, economic and development levels, and meat-eating habits might have positive impacts on the intake of dietary zinc. A lower consumption of grains and a higher consumption of livestock meat were characteristic of the groups with relatively better dietary zinc intakes. An adequate dietary zinc intake was associated with reduced diastolic blood pressure and fasting glucose levels in Chinese women, but with raised TG in Chinese adults. Nutrition interventions should be strengthened for Chinese men and lower socioeconomic groups.

## Figures and Tables

**Table 1:** Sample characteristics of the study population aged 18–64 years in the China Nutritional Transition Cohort Survey (CNTCS).

Group	Male	Female	Total
**Total**	5665(47.1)	6363(52.9)	12028(100.0)
**Age, %**			
18–49	3127(55.2)	3561(56.0)	6688(55.6)
50–64	2538(44.8)	2802(44.0)	5340(44.4)
**Region, %**			
Country	2520(44.5)	2773(43.6)	5293(44.0)
County	1029(18.2)	1144(18)	2173(18.1)
Suburb	948(16.7)	1064(16.7)	2012(16.7)
City	1168(20.6)	1382(21.7)	2550(21.2)
**Education, %**			
Primary/below	1126(19.9)	1929(30.3) ^#^	3055(25.4)
Middle school	2078(36.7)	2101(33.0)	4179(34.7)
High school	1509(26.6)	1386(21.8)	2895(24.1)
University/above	952(16.8)	947(14.9)	1899(15.8)
**Income, %**			
low	1804(31.8)	2196(34.5) ^#^	4000(33.3)
Medium	1912(33.8)	2088(32.8)	4000(33.3)
High	1949(34.4)	2079(32.7)	4028(33.5)
**Area, %**			
Western	2006(35.4)	2246(35.3)	4252(35.4)
Central	1511(26.7)	1676(26.3)	3187(26.5)
Eastern	2148(37.9)	2441(38.4)	4589(38.2)
**Smoke, %**			
No	2454(43.3)	6251(98.2)	8705(72.4)
Yes	3211(56.7)	112(1.8)	3323(27.6)
**Alcohol, %**			
No	2494(44.0)	5928(93.2)	8422(70.0)
Yes	3171(56.0)	435(6.8)	3606(30.0)
**BMI, kg/m^2^**	24.4 ± 3.7	24.0 ± 4.2 *	24.2 ± 4.0
**Energy, kcal/day**	2179.8 ± 733.3	1841.9 ± 642.0 *	2001.1 ± 706.9
**Protein, g/day**	67.6 ± 25.5	57.0 ± 22.0 *	62.0 ± 24.3
**Carbohydrate, g/day**	276.7 ± 119.6	236.7 ± 103.1 *	255.6 ± 113
**Fat, g/day**	86.6 ± 44.7	73.8 ± 40.2 *	79.8 ± 42.9
**Systol blood presure, mmHg**	127.4 ± 16.8	122.8 ± 18.1 *	124.9 ± 17.7
**Diastol blood presure, mmHg**	82.8 ± 11	78.9 ± 10.8 *	80.7 ± 11.1
**Fasting glucose, mmol/L**	5.5 ± 1.6	5.3 ± 1.3 *	5.4 ± 1.5
**TG, mmol/L**	4.9 ± 1.1	4.9 ± 1.1	4.9 ± 1.1
**Na, mg/day**	5330.2 ± 5419	4638.1 ± 5036.8 *	4964.1 ± 5231.5

^#^: Chi square trend test, compared with female, *p* < 0.001. *: Analysis of variance, compared with female, *p* < 0.001. BMI, body mass index; TG, Triglyceride. BMI, energy, protein, carbohydrate, fat, systolic blood pressure, diastolic blood pressure, fasting glucose, TG and sodium are described as means ± SD.

**Table 2:** Mean of zinc intake and zinc density by gender, age, region, education, income, and area subgroups.

	Zinc Intake (mg/day) (means ± SD)			Zinc Density (mg/day/1000 kcal) (means ± SD)		
Group	Male	Female	Total	Male	Female	Total
**Total**	11.2 ± 4.2	9.4 ± 3.6	10.2 ± 4.0	5.2 ± 1.3	5.2 ± 1.3	5.2 ± 1.3
*p*-values for difference ^†^		<0.001 *				
**Age**						
18–49	11.4 ± 4.3	9.6 ± 3.7	10.4 ± 4.1	5.3 ± 1.3	5.2 ± 1.3	5.3 ± 1.3
50–64	10.9 ± 4.0	9.2 ± 3.5	10.0 ± 3.9	5.2 ± 1.3	5.1 ± 1.2	5.1 ± 1.3
*p*-values for difference ^†^	<0.001	<0.001	<0.001	<0.05	<0.001	<0.001
**Region**						
Country	11.0 ± 4.2	9.2 ± 3.6	10.1 ± 4.0	5.1 ± 1.2	5.0 ± 1.2	5.1 ± 1.2
County	11.0 ± 4.1	9.3 ± 3.3	10.1 ± 3.8	5.2 ± 1.2	5.2 ± 1.2	5.2 ± 1.2
Suburb	11.2 ± 4.3	9.5 ± 3.9	10.3 ± 4.1	5.4 ± 1.4	5.3 ± 1.4	5.3 ± 1.4
City	11.5 ± 4.1	9.8 ± 3.8	10.6 ± 4.0	5.4 ± 1.4	5.4 ± 1.4	5.4 ± 1.4
*p*-values for linear trend ^‡^	<0.001	<0.001	<0.001	<0.001	<0.001	<0.001
**Education**						
Primary/below	11.0 ± 3.9	9.2 ± 3.4	9.9 ± 3.7	5.2 ± 1.4	5.1 ± 1.2	5.1 ± 1.3
Middle school	11.1 ± 4.3	9.4 ± 3.8	10.3 ± 4.2	5.2 ± 1.3	5.1 ± 1.3	5.1 ± 1.3
High school	11.2 ± 4.2	9.5 ± 3.6	10.4 ± 4.0	5.2 ± 1.3	5.3 ± 1.3	5.2 ± 1.3
University/above	11.5 ± 4.2	9.6 ± 3.6	10.6 ± 4.0	5.5 ± 1.3	5.4 ± 1.3	5.5 ± 1.3
*p*-values for linear trend ^‡^	<0.05	<0.05	<0.001	<0.001	<0.001	<0.001
**Income**						
low	11.1 ± 4.2	9.5 ± 3.8	10.2 ± 4.1	5.1 ± 1.3	5.1 ± 1.3	5.1 ± 1.3
Medium	10.9 ± 4.1	9.2 ± 3.5	10.0 ± 3.9	5.2 ± 1.3	5.2 ± 1.2	5.2 ± 1.3
High	11.5 ± 4.2	9.5 ± 3.6	10.5 ± 4.0	5.4 ± 1.3	5.3 ± 1.3	5.3 ± 1.3
*p*-values for linear trend ^‡^	<0.001	<0.001	<0.001	<0.001	<0.001	<0.001
**Area**						
Western	11.3 ± 4.3	9.7 ± 3.9	10.5 ± 4.1	5.2 ± 1.3	5.2 ± 1.3	5.2 ± 1.3
Central	11.0 ± 4.1	9.1 ± 3.5	10.0 ± 3.9	5.0 ± 1.3	5.0 ± 1.2	5.0 ± 1.2
Eastern	11.1 ± 4.1	9.3 ± 3.5	10.2 ± 3.9	5.3 ± 1.3	5.3 ± 1.3	5.3 ± 1.3
*p*-values for linear trend ^‡^		<0.001	<0.001	<0.001	<0.001	<0.001

*: Compared with male; values adjusted for age, region, education, income, and area, the intakes of energy, protein, carbohydrates and fats; ^†^: *p*-values from analysis of variance (ANOVA); ^‡^: *p*-values from linear regression. The zinc intake in the age, region, education, income, and area groups was adjusted for energy, protein, carbohydrates, and fats; The zinc density in the above group was adjusted for protein, carbohydrates, and fats.

**Table 3:** Distribution of dietary zinc intake levels by gender, age, region, education, income, and area subgroups (%).

	Male				Female			
Group	*N*	<EAR	EAR-RNI	≥RNI	*N*	<EAR	EAR-RNI	≥RNI
**Total**	5665	49.2	20.1	30.7	6363	14.8	18.3	67.0
*p*-values for difference ^†^						<0.05 *		<0.05 *
**Age**								
18–49	3127	45.9	20.7	33.5	3561	13.8	17.4	68.8
50–64	2538	53.3	19.4	27.3	2802	16.1	19.3	64.6
*p*-values for difference ^†^		<0.05		<0.05		<0.05		<0.05
**Region**								
Country	2520	50.3	19.9	29.8	2773	16.7	19.1	64.3
County	1029	50.2	19.8	29.9	1144	15.1	15.9	69.0
Suburb	948	49.2	20.4	30.5	1064	14.7	18.6	66.7
City	1168	45.9	20.5	33.7	1382	10.8	18.4	70.8
*p*-values for trend ^‡^		<0.05		<0.05		<0.05		<0.05
**Education**								
Primary/below	1126	50.1	20.7	29.2	1929	16.2	18.1	65.7
Middle school	2078	49.8	20.0	30.2	2101	15.3	19.0	65.7
High school	1509	50.3	19.8	29.9	1386	14.1	18.0	68.0
University/above	952	45.1	20.0	35.0	947	11.8	17.4	70.8
*p*-values for trend ^‡^				<0.05		<0.05		<0.05
**Income**								
low	1804	50.9	19.2	29.9	2196	15.3	18.0	66.8
Medium	1912	51.1	19.6	29.3	2088	16.9	18.3	64.9
High	1949	45.8	21.4	32.8	2079	12.2	18.6	69.3
*p*-values for trend ^‡^		<0.05		0.0497		<0.05		
**Area**								
Western	2006	47.5	19.2	33.3	2246	14.3	15.7	70.1
Central	1511	50.4	20.3	29.3	1676	16.8	20.3	63.0
Eastern	2148	49.9	20.7	29.4	2441	13.9	19.3	66.8
*p*-values for trend ^‡^				<0.05		<0.05	<0.05	<0.05

*: compared with male; ^†^: *p*-values from Chi-squared test; ^‡^: *p*-values from Cochran-Armitage trend test. Dietary zinc intake levels: <EAR: less than the Estimated Average Requirement (EAR); EAR-RNI: equal to or greater than the EAR and less than the recommended nutrient intake (RNI); ≥RNI: equal to or greater than the RNI.

**Table 4:** Food sources of dietary zinc intake by gender, age, region, education, income, and area (%).

Group	Grains	Livestock Meat	Vegetables	Legumes	Seafood	Other
**Total**	38.8	18.7	9.5	6.1	4.7	22.2
**Gender**						
Male	39.2	19.6	9.3	6.0	4.8	21.1
Female	38.4	17.8	9.8	6.3	4.7	23.0
*p*-values for difference ^†^	<0.001	<0.001	<0.001	<0.001	<0.001	
**Age**						
18–49	38.3	19.8	9.2	5.8	4.7	22.1
50–64	39.3	17.4	9.9	6.6	4.7	22.0
*p*-values for difference ^†^	<0.001	<0.001	<0.001	<0.001		
**Region**						
Country	44.2	15.3	10.3	6.9	3.9	19.5
County	39.7	18.9	9.5	5.9	5.1	21.0
Suburb	36.0	20.5	10.1	6.1	4.7	22.7
City	31.2	23.0	8.1	5.2	5.7	26.8
*p*-values for trend ^‡^	<0.001	<0.001	<0.001	<0.001	<0.001	
**Education**						
Primary/below	44.7	14.4	10.8	7.2	3.7	19.2
Middle school	40.8	17.7	9.9	6.6	4.2	20.8
High school	36.2	20.4	9.1	5.7	5.1	23.5
University/above	31.9	23.2	8.0	4.9	6.2	25.8
*p*-values for trend ^‡^	<0.001	<0.001	<0.001	<0.001	<0.001	
**Income**						
low	42.7	16.7	9.3	6.6	3.9	20.8
Medium	40.7	16.6	10.6	6.5	4.5	21.0
High	35.1	21.4	9.0	5.6	5.3	23.7
*p*-values for trend ^‡^	<0.001	<0.001	<0.001	<0.001	<0.001	
**Area**						
Western	34.1	30.4	9.8	3.3	3.8	18.6
Central	43.4	17.4	11.5	6.2	3.1	18.4
Eastern	36.0	18.8	8.2	6.3	5.8	24.9
*p*-values for trend ^‡^	<0.001	<0.001	<0.001	<0.001	<0.001	

^†^: *p*-values from Chi-squared test; ^‡^: *p*-values from Cochran–Armitage trend test.

**Table 5:** Relationship between dietary zinc intake levels and metabolic syndrome indicators for males.

	β	SE	*t*	*P*
**systolic blood pressure**				
<EAR	ref			
EAR-RNI	0.506	0.4763	1.06	0.288
≥RNI	0.280	0.570	1.49	0.623
**diastolic blood pressure**				
<EAR	ref			
EAR-RNI	0.030	0.318	0.10	0.924
≥RNI	−0.366	0.380	−0.96	0.335
**fasting glucose**				
<EAR	ref			
EAR-RNI	−0.120	0.067	−1.78	0.075
≥RNI	−0.155	0.080	−1.93	0.054
**TG**				
<EAR	ref			
EAR-RNI	0.083	0.063	1.31	0.190
≥RNI	0.219	0.076	2.90	0.004

**Table 6:** Relationship between dietary zinc intake levels and metabolic syndrome indicators for females.

	β	SE	*t*	*P*
**systolic blood pressure**				
<EAR	ref			
EAR-RNI	0.268	0.550	0.49	0.627
≥RNI	0.413	0.546	0.76	0.450
**diastolic blood pressure**				
<EAR	ref			
EAR-RNI	−0.011	0.344	−0.03	0.976
≥RNI	−0.963	0.341	−2.82	0.005
**fasting glucose**				
<EAR	ref			
EAR-RNI	−0.052	0.063	−0.82	0.411
≥RNI	−0.187	0.063	−2.98	0.003
**TG**				
<EAR	ref			
EAR-RNI	0.097	0.043	2.24	0.025
≥RNI	0.120	0.042	2.81	0.005

## References

[1] Gibson R.S., Ferguson E.L. (2015). An Interactive 24-Hour Recall for Assessing the Adequacy of Iron and Zinc Intakes in Developing Countries.

[2] Zhai F.-Y., Yang X.G. (2006). Report on Nutrition and Health Status of Chinese Residents—Dietary and Nutrient Intake in 2002.

[3] Liu S., Li J., Song Y., Gong C.-R., Cheng M.-W. (2017). Dietary intake of zinc and its changing trend among residents in Hubei Province, 1991–2011. Pract. Prev. Med..

[4] Du W., Wang H., Chen S., Su C., Zhang H., Zhang B. (2015). Trend of dietary nutrient intake among adult females in 9 provinces in China, 2000–2011. Chin. J. Epidemiol..

[5] Zhang J.-G., Zhang B., Wang H.-J., Wang Z.-H., Du W.-W., Su C., Zhang J., Zhai F.-Y. (2012). Zinc intake trend of Chinese adults aged 50–79 in nine provinces (autonomous regions) from 1991 to 2009. Chin. J. Health Educ..

[6] Zhang J.-G., Zhang B., Wang H.-J., Du W.-W., Su C., Zhai F.-Y. (2012). Nutrients intake trend of Chinese population in nine provinces from 1989-2009 (VII) Zinc intake trend of Chinese adults aged 18–49 years. Acta Nutr. Sin..

[7] Wang Z.-H., Zhai F.-Y., He Y.-N., Hu Y.-S., Wang H.-J. (2006). Dietary zinc intake of Chinese residents and the trend of change. J. Hyg. Res..

[8] Yu D.M., He Y.N., Guo Q.Y., Fang H.Y., Xu X.L., Fang Y.H., Li J., Zhao L.Y. (2016). Trends of energy and nutrients intake among Chinese population in 2002–2012. J. Hyg. Res..

[9] King J.C., Brown K.H., Gibson R.S., Krebs N.F., Lowe N.M., Siekmann J.H., Raiten D.J. (2016). Biomarkers of nutrition for development (bond)-zinc review. J. Nutr..

[10] King J.C., Cousins R.J., Shils M.E., Shike M., Ross A.C., Caballero B., Cousins R.J. (2006). Zinc. Modern Nutrition in Health and Disease.

[11] Prasad A.S. (1984). Discovery and importance of zinc in human nutrition. Fed. Proc..

[12] Samman S. (2007). Zinc. Nutr. Diet..

[13] Prasad A.S. (1995). Zinc: An overview. Nutrition.

[14] Vallee B.L., Falchuk K.H. (1993). The biochemical basis of zinc physiology. Physiol. Rev..

[15] Powell S.R. (2000). The antioxidant properties of zinc. J. Nutr..

[16] Hennig B., Wang Y., Ramasamy S., McClain C.J. (1992). Zinc deficiency alters barrier function of cultured porcine endothelial cells. J. Nutr..

[17] Freitas E.P., Cunha A.T., Aquino S.L., Pedrosa L.F., Lima S.C., Lima J.G., Almeida M.G., Sena-Evangelista K. (2017). Zinc Status Biomarkers and Cardiometabolic Risk Factors in Metabolic Syndrome: A Case Control Study. Nutrients.

[18] Olechnowicz J., Tinkov A., Skalny A., Suliburska J. (2018). Zinc status is associated with inflammation, oxidative stress, lipid, and glucose metabolism. J. Physiol. Sci..

[19] Trame S., Wessels I., Haase H., Rink L. (2018). A short 18 items food frequency questionnaire biochemically validated to estimate zinc status in humans. J. Trace Elem. Med. Biol..

[20] Zhang B., Zhai F.Y., Du S.F., Popkin B.M. (2014). The China Health and Nutrition Survey, 1989–2011. Obesity Rev..

[21] Yang Y., Wang G., Pan X. (2009). Chinese Food Composition Table.

[22] Chinese Nutrition Society (2014). Chinese Dietary Reference Intakes 2013.

[23] Zhang Z.-Z. (2010). Evolution and Evaluation of the Chinese Economic Regions Division. J. Shanxi Univ. Financ. Econ. (High. Educ. Ed.).

[24] (2016). China’s Nutrition and Health Status Monitoring Comprehensive Report in 2010–2013.

[25] Ma G., Li Y., Jin Y., Du S., Kok F.J., Yang X. (2007). Assessment of intake inadequacy and food sources of zinc of people in China. Public Health Nutr..

[26] National Health and Family Planning Commission Disease Prevention and Control Bureau (2016). Report on Nutrition and Chronic Diseases in China (2015 Report).

[27] Lim K.H., Riddell L.J., Nowson C.A., Booth A.O., Szymlek-Gay E.A. (2013). Iron and Zinc Nutrition in the Economically-Developed World: A Review. Nutrients.

[28] Moshfegh A., Goldman J., Cleveland L. (2005). What We Eat in America, NHANES 2001–2002: Usual Nutrient Intakes from Food Compared to Dietary Reference Intakes.

[29] O’Neil C.E., Keast D.R., Fulgoni V.L., Nicklas T.A. (2012). Food sources of energy and nutrients among adults in the US: NHANES 2003–2006. Nutrients.

[30] US Department of Agriculture, Agricultural Research Service (2010). 2009–2010 What We Eat in America, NHANES Tables 1–40. http://www.ars.usda.gov/SP2UserFiles/Place/12355000/pdf/0910/tables_1-40_2009-2010.pdf.

[31] Statistics Canada (2009). Canadian Community Health Survey Cycle 2.2, Nutrition 2004.

[32] University of Otago and Ministry of Health (2011). A Focus on Nutrition: Key Findings of the 2008/09 New Zealand Adult Nutrition Survey.

[33] Henderson L., Irving K., Gregory J., Bates C., Prentice A., Perks J., Swan G., Farron M. (2003). The National Diet and Nutrition Survey: Adults Aged 19 to 64 Years—Vitamin and Mineral Intake and Urinary Analytes.

[34] UK Department of Health (2011). National Diet and Nutrition Survey: Headline Results from Years 1 and 2 (Combined) of the Rolling Programme, 2008/09–2009/10; Headline Results from Years 1 and 2 (Combined) Tables. https://www.gov.uk/government/uploads/system/uploads/attachment_data/file/152237/dh_128556.pdf.

[35] Olivares M., Pizarro F., de Pablo S., Araya M., Uauy R. (2004). Iron, zinc, and copper: Contents in common Chilean foods and daily intakes in Santiago, Chile. Nutrition.

[36] Ma G., Li Y., Jin Y., Zhai F., Kok F.J., Yang X. (2007). Phytate intake and molar ratios of phytate to zinc, iron and calcium in the diets of people in China. Eur. J. Clin. Nutr..

[37] Tubek S. (2007). Role of zinc in regulation of arterial blood pressure and in the etiopathogenesis of arterial hypertension. Biol. Trace Elem. Res..

[38] Tomat A.L., Weisstaub A.R., Jauregui A., Piñeiro A., Balaszczuk A.M., Costa M.A., Arranz C.T. (2005). Moderate zinc deficiency influences arterial blood pressure and vascular nitric oxide pathway in growing rats. Pediatr. Res..

[39] Bergomi M., Rovesti S., Vinceti M., Vivoli R., Caselgrandi E., Vivoli G. (1997). Zinc and copper status and blood pressure. J. Trace Elem. Med. Biol..

[40] Sato M., Kurihara N., Moridaira K., Sakamoto H., Tamura J., Wada O., Yanagisawa H. (2003). Dietary Zn deficiency does not influence systemic blood pressure and vascular nitric oxide signaling in normotensive rats. Biol. Trace Elem. Res..

[41] Kurihara N., Yanagisawa H., Sato M., Tien C.K., Wada O. (2002). Increased renal vascular resistance in zinc-deficient rats: Role of nitric oxide and superoxide. Clin. Exp. Pharmacol. Physiol..

[42] Taittonen L., Nuutinen M., Räsänen L., Mussalo-Rauhamaa H., Turtinen J., Uhari M. (1997). Lack of association between copper, zinc, selenium and blood pressure among healthy children. J. Hum. Hypertens..

[43] Aydemir T.B., Chang S.M., Guthrie G.J., Maki A.B., Ryu M.S., Karabiyik A., Cousins R.J. (2012). Zinc transporter ZIP14 functions in hepatic zinc, iron and glucose homeostasis during the innate immune response (endotoxemia). PLoS ONE.

[44] Sinha S., Sen S. (2014). Status of zinc and magnesium levels in type 2 diabetes mellitus and its relationship with glycemic status. Int. J. Diabetes Dev. Ctries..

[45] Olaniyan O.O., Awonuga M.A.M., Ajetunmobi A.F., Adeleke I.A., Fagbolade O.J., Olabiyi K.O., Oyekanmi B.A., Osadolor H.B. (2012). Serum copper and zinc levels in Nigerian type 2 diabetic patients. Afr. J. Diabetes Med..

[46] Devi T.R., Hijam D., Dubey A., Debnath S., Oinam P., Devi N.G.T., Singh W.G. (2016). Study of serum zinc and copper levels in type 2 diabetes mellitus. Int. J. Contemp. Med. Res..

[47] Kaur J., Singh T. (2015). Estimation of serum magnesium and zinc levels in type-2 diabetes mellitus. Int. J. Bioassays.

[48] Yahya H., Yahya K.M., Saqib A. (2011). Minerals and type 2 diabetes mellitus—Levels of zinc, magnesium and chromium in diabetic and nondiabetic population. Univ. Med. Dent. Coll..

[49] Jyothirmayi B., Vasantha M. (2015). Study of zinc and glycated Hb levels in diabetic complications. Int. J. Pharm. Clin. Res..

[50] Kumar D.A., Priya V.S., Jaiprabhu J., Ramalingam K. (2014). Serum copper and zinc levels significance in type 2 diabetic patients. J. Med. Sci. Technol..

[51] Bao B., Prasad A.S., Beck F.W.J., Fitzgerald J.T., Snell D., Bao G.W., Singh T., Cardozo L.J. (2010). Zinc decreases C-reactive protein, lipid peroxidation, and inflammatory cytokines in elderly subjects: A potential implication of zinc as an atheroprotective agent. Am. J. Clim. Nutr..

[52] Ho M., Baur L.A., Cowell C.T., Samman S., Garnett S.P. (2017). Zinc status, dietary zinc intake and metabolic risk in Australian children and adolescents; Nepean Longitudinal Study. Eur. J. Nutr..

[53] El-Ashmony S.M.A., Morsi H.K., Abdelhafez A.M. (2012). Effect of zinc supplementation on glycemic control, lipid profile, and renal functions in patients with type II diabetes: A single blinded, randomized, placebo-controlled, trial. J. Biol. Agric. Health.

[54] Kanoni S., Nettleton J.A., Hivert M.-F., Ye Z., van Rooij F.J.A., Shungin D., Sonestedt E., Ngwal J.S., Wojczynski M.K., Lemaitre R.N. (2011). Total zinc intake may modify the glucose-raising effect of a zinc transporter (SLC30A8) variant a 14-cohort meta-analysis. Diabetes.

[55] Islam M.R., Attia J., Ali L., McEvoy M., Selim S., Sibbritt D., Akhter A., Akter S., Peel R., Faruque O. (2016). Zinc supplementation for improving glucose handling in pre-diabetes: A double-blind randomized placebo controlled pilot study. Diabetes Res. Clin. Pract..

[56] Karamali M., Heidarzadeh Z., Seifati S.M., Samimi M., Tabassi Z., Hajijafari M., Asemi Z., Esmaillzadeh A. (2015). Zinc supplementation and the effects on metabolic status in gestational diabetes: A randomized, double-blind, placebo-controlled trial. J. Diabetes Complic..

[57] Jansen J., Rosenkranz E., Overbeck S., Warmuth S., Mocchegiani E., Giacconi R., Weiskirchen R., Karges W., Rink L. (2012). Disturbed zinc homeostasis in diabetic patients by in vitro and in vivo analysis of insulinomimetic activity of zinc. J. Nutr. Biochem..

[58] Payahoo L., Ostadrahimi A., Mobasseri M., Bishak Y.K., Farrin N., Jafarabadi M.A., Ostadrahimi A. (2013). Effects of zinc supplementation on the anthropometric measurements, lipid profiles and fasting blood glucose in the healthy obese adults. Adv. Pharm. Bull..

[59] El Dib R., Gameiro O.L., Ogata M.S., Mo’dolo N.S., Braz L.G., Jorge E.C., do Nascimento P., Beletate V. (2015). Zinc supplementation for the prevention of type 2 diabetes mellitus in adults with insulin resistance. Cochrane Database Syst. Rev..

[60] Ranasinghe P., Wathurapatha W.S., Ishara M.H., Jayawardana R., Galappatthy P., Katulanda P., Constantine G.R. (2015). Effects of Zinc supplementation on serum lipids: A systematic review and meta-analysis. Nutr. Metab..

[61] Li H.T., Jiao M., Chen J., Liang Y. (2010). Roles of zinc and copper in modulating the oxidative refolding of bovine copper, zincsuperoxide dismutase. Acta Biochim. Biophys. Sin. (Shanghai).

[62] Tayeb W., Nakbi A., Cheraief I., Miled A., Hammami M. (2013). Alteration of lipid status and lipid metabolism, induction of oxidative stress and lipid peroxidation by 2,4-dichlorophenoxyacetic herbicide in rat liver. Toxicol. Mech. Methods.

[63] Li X., Guan Y., Shi X., Ding H., Song Y., Li C., Liu R., Liu G. (2013). Effects of high zinc levels on the lipid synthesis in rat hepatocytes. Biol. Trace Elem. Res..

[64] Weigand E., Egenolf J. (2017). Moderate zinc deficiency does not alter lipid and fatty acid composition in the liver of weanling rats fed diets rich in cocoa butter or safflower oil. J. Nutr. Metab..

[65] Khan M.I., Siddique K.U., Ashfaq F., Ali W., Reddy H.D., Mishra A. (2013). Effect of high-dose zinc supplementation with oral hypoglycemic agents on glycemic control and inflammation in type-2 diabetic nephropathy patients. J. Nat. Sci. Biol. Med..

[66] Gunasekara P., Hettiarachchi M., Liyanage C., Lekamwasam S. (2011). Effects of zinc and multimineral vitamin supplementation on glycemic and lipid control in adult diabetes. Diabetes Metab. Syndr. Obes..

[67] Seo J.A., Song S.W., Han K., Lee K.J., Kim H.N. (2010). The associations between serum zinc levels and metabolic syndrome in the Korean population: Findings from the 2010 Korean National Health and Nutrition Examination Survey. PLoS ONE.

[68] Jihye K. (2013). Dietary zinc intake is inversely associated with systolic blood pressure in young obese women. Nutr. Res. Pract..

[69] Lowe N.M., Medina M.W., Stammers A.L., Patel S., Souverein O.W., Dullemeijer C., Serra-Majem L., Nissensohn M., Moran V.H. (2012). The relationship between zinc intake and serum/plasma zinc concentration in adults: A systematic review and dose–response meta-analysis by the EURRECA Network. Br. J. Nutr..

[70] Pang S.J., Jia S.S., Man Q.Q., Song S., Li Y.Q., Song P.K., Zhao W.H., Zhang J. (2017). Dietary Cholesterol in the Elderly Chinese Population: An Analysis of CNHS 2010–2012. Nutrients.

[71] Ma G., Cui Z., Li Y., Hu X., Wang J., Yang X. (2006). The survey about the use of dietary supplements by Chinese Adults. Acta Nutr. Sin..

[72] Briefel R.R., Bialostosky K., Kennedy-Stephenson J., McDowell A.M., Ervin R.B., Wright J.D. (2000). Zinc intake of the US population: Findings from the Third National Health and Nutrition Examination Survey, 1988–1994. J. Nutr..

